# Pulse Oximetry Values in Newborns with Critical Congenital Heart Disease upon ICU Admission at Altitude

**DOI:** 10.3390/ijns4040030

**Published:** 2018-10-31

**Authors:** John S. Kim, Merlin W. Ariefdjohan, Marci K. Sontag, Christopher M. Rausch

**Affiliations:** 1 Department of Pediatrics, Heart Institute, Children’s Hospital Colorado, University of Colorado School of Medicine, Aurora, CO 80045, USA; 2 Department of Epidemiology, Colorado School of Public Health, University of Colorado Anschutz Medical Campus, Aurora, CO 80045, USA; 3 Center for Public Health Innovation at CI International, Littleton, CO 80120, USA

**Keywords:** critical congenital heart disease, pulse oximetry, newborn screening, altitude

## Abstract

Pulse oximetry screening for critical congenital heart disease (CCHD) has been recommended by the American Academy of Pediatrics (AAP). The objectives of this study are to describe saturation data, and to evaluate the effectiveness of AAP-recommended pulse oximetry screening guidelines applied retrospectively to a cohort of newborns with known CCHD at moderate altitude (5557 feet, Aurora, Colorado). Data related to seven critical congenital heart disease diagnoses were extracted from electronic health records (pulse oximetry, prostaglandin administration, and oxygen supplementation). Descriptive epidemiologic data were calculated. 158 subjects were included in this analysis; the AAP pulse oximetry screening protocol was applied to 149 subjects. Mean pre-ductal and post-ductal pulse oximetry values of the infants known to have CCHD at 24 h of life were 87.1% ± 7.2 and 87.8% ± 6.3, respectively. Infants treated with prostaglandins and oxygen had lower oximetry readings. The screening algorithm would have identified 80.5% of infants with known CCHDs (120/149 subjects). Additionally, sequential pulse oximetry screening based on the AAP-recommended protocol was able to identify a true positive screen capture rate of 80.5% at moderate altitude.

## 1. Introduction

Congenital heart disease is among the most common birth defects, with an incidence of approximately 1 per 100 live births. Critical congenital heart disease (CCHD) is defined as a structural heart defect with significant risk for mortality without intensive intervention. CCHD occurs in approximately 4 per 1000 live births, and it is estimated that 13–55% of newborns with CCHD are discharged from hospital undiagnosed [1,2,3].

Screening with pulse oximetry has been identified as a low-cost non-invasive test that improves the ability to diagnose CCHD when compared to physical examination alone [4,5]. Several CCHD lesions are particularly amenable to identification via pulse oximetry screening, including truncus arteriosus (TA), transposition of the great arteries (TGA), tricuspid valve atresia (TVA), tetralogy of Fallot (TOF), total anomalous pulmonary venous return (TAPVR), hypoplastic left heart syndrome (HLHS), and pulmonary valve atresia with intact ventricular septum (PA/IVS) [6,7,8]. The most widely utilized pulse oximetry screening guidelines recommend initiation at or beyond 24 h of life with measurement of pre- and post-ductal saturations (right hand and foot, respectively) [8]. Pulse oximetry screening in the first 24–48 h after birth has been found to be highly specific (99.9% specificity; 95% confidence interval (CI) 99.7, 99.9), but with a lower sensitivity of 76.3% (95% CI 69.5, 82.0) in one meta-analysis [9].

The performance of pulse oximetry screening has been evaluated in many studies performed at sea level [10], however, studies at altitude are limited [3,11]. Evaluation of the effectiveness of pulse oximetry screening guidelines is complicated due to the small number of infants likely to be identified through newborn screening as many infants with CCHD are diagnosed prenatally or in the first 24 h of life, thereby reducing the potential number of infants with undiagnosed CCHD eligible for screening with pulse oximetry. Furthermore, studies of newborn oxygen saturations have suggested a wider range and lower average saturations for infants born at higher altitude, further complicating evaluation of the utility of pulse oximetry in this population [12,13,14,15]. We retrospectively reviewed pulse oximetry values for a population of newborns between 24 and 48 h of life who were known to have CCHD to provide an epidemiologic description of saturation data in newborns with CCHD at altitude.

## 2. Materials and Methods

We performed a retrospective cohort study at a regional tertiary children’s hospital at moderate altitude (5557 feet, Aurora, CO, USA). We queried the electronic health record (EHR) for newborns presenting within the first 24–48 h of life with one of the 7 CCHD diagnoses using International Classification of Disease (ICD) codes in the years 2006–2013 (i2b2; Informatics for Integrating Biology and the Bedside, Version 1.7, Partners HealthCare System, Boston, MA, USA). Subjects included both prenatally and postnatally diagnosed CCHD. We reviewed the EHR for confirmation of diagnosis, pulse oximetry readings, prostaglandin (PGE) administration, and oxygen supplementation data at 24, 28, 36, and 48 h (±2 h) of life. These time points were within the American Academy of Pediatrics (AAP)-recommended window of screening between 24 and 48 h [8]. Pulse oximetry was measured continuously in all subjects with simultaneous pre- and post-ductal saturation values recorded in the EHR. Pulse oximetry values were recorded hourly in the EHR via standard clinical practice upon bedside nurse confirmation of the plethysmograph waveform during the patient’s usual calm physiologic state.

In a secondary analysis of the pulse oximetry data, we applied a simulated pulse oximetry screening protocol modeled after the recommended AAP algorithm [8]. Pulse oximetry values were categorized based on the AAP screening protocol: (1) passed screen (no concern for CCHD, false negative): saturation ≥95% and ≤3% difference between pre- and post-ductal readings; (2) borderline reading (to be repeated at next time point): saturation 90–94% and/or >3% difference between pre- and post-ductal readings; (3) failed screen (concern for CCHD, true positive): saturation <90% in either pre- or post-ductal reading. Up to 3 total hypothetical screens were applied to the retrospective data collected at times closest to 28, 36, and 48 h of life. Data that resulted in a positive or negative screen for CCHD (based on the AAP protocol) were not evaluated in subsequent time points. Subjects with borderline pulse oximetry values at 3 consecutive readings were considered to meet screening criteria for concern for CCHD. Since all participating subjects had a CCHD diagnosis, subjects in whom pulse oximetry readings raised no concern for CCHD were considered false negative in this hypothetical screening. All other subjects were considered true positives. This secondary analysis allowed us to estimate the general effectiveness of the AAP protocol (i.e., false negative vs. true positive) in a cohort of infants with known CCHD at altitude based on retrospective data.

Study data were collected and maintained by using Research Electronic Data Capture (REDCap) tools hosted at the University of Colorado [16]. This study was approved by the Colorado Multiple Institutional Review Board with a full waiver of Health Insurance and Portability and Accountability Act (HIPAA) authorization.

Baseline demographic data are presented as means, standard deviations, and range. Group proportions for categorical variables were statistically assessed using the Fisher exact test. The Shapiro-Wilk test for normality was used to evaluate distributions for all continuous variables. Then, the *t*-test and Wilcoxon-Mann-Whitney test were applied to compare continuous variables between groups for those with normal and non-normal distributions, respectively. Pre- and post-ductal pulse oximetry values collected at 24, 28, 36, and 48 h were summarized as a box-whisker plot showing the mean, median, minimum/maximum values, and interquartile ranges. Mean pulse oximetry readings for various types of clinical support (i.e., PGE, oxygen, both, or none) were compared to each other using the Kruskal-Wallis test due to non-normality of data distribution. Post-hoc analysis was made on groups showing statistical difference. Statistical significance level was set at *p* < 0.05 and 95% CI were calculated for mean pulse oximetry values. All analyses were performed using SAS software (version 9.4; SAS Institute, Cary, NC, USA).

## 3. Results

### 3.1. Descriptive Analysis of Study Cohort

**Figure 1:**
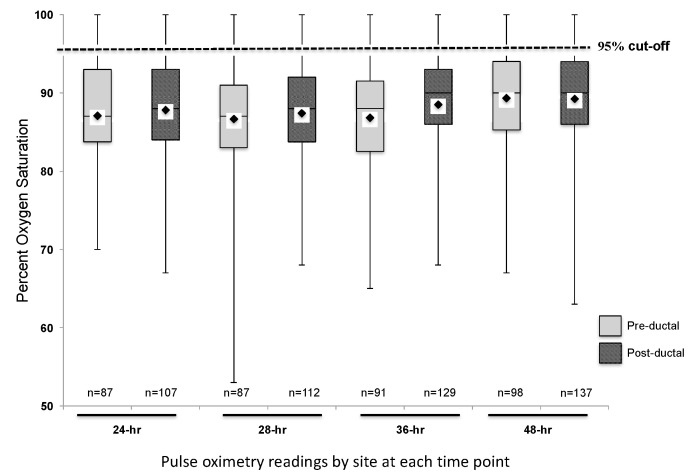
Pre- and post-ductal pulse oximetry data at 24, 28, 36, and 48 h of life (*n* = 158). Mean saturation at each time point indicated by the diamond on each box plot.

**Figure 2:**
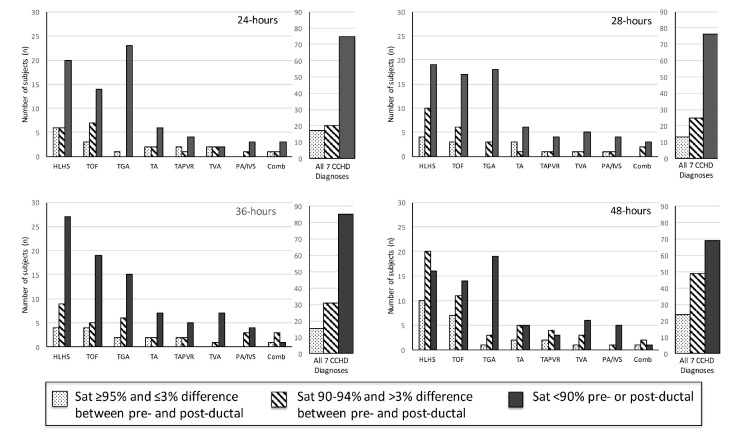
Pulse oximetry by saturation group at 24, 28, 36, and 48 h of life (*n* = 158).

Two hundred and fourteen subjects with CCHD were identified. Fifty-six subjects were excluded from data collection due to the following reasons: insufficient pulse oximetry data in the EHR, death prior to 24 h of life, or atrial septostomy or surgery prior to 24 h of life. One hundred and fifty-eight subjects were included in this analysis. The distribution of CCHD anatomic diagnoses between the subjects included and excluded from the study was different (Table 1), but we found no other differences between the groups. The mean pre-ductal pulse oximetry readings at 24, 28, 36, and 48 h were 87.1 ± 7.2% (95% CI 85.6, 88.6), 86.7 ± 7.2% (85.2, 88.2), 86.8 ± 7.6% (85.2, 88.4), and 89.3 ± 6.5% (88.0, 90.6), respectively. The mean post-ductal pulse-oximetry readings were 87.8 ± 6.3% (95% CI 86.6, 89.0), 87.4 ± 7.3% (86.1, 88.8), 88.5 ± 6.7% (87.3, 89.7), and 89.2 ± 6.3% (88.2, 90.3), respectively. Figure 1 summarizes the overall distribution of pre- and post-ductal saturation readings at the four time points. For descriptive purposes, data were also stratified by diagnoses and pulse oximetry readings for each collection time (Figure 2). To evaluate the effects of treatment on pulse oximetry, we compared pulse oximetry data in infants receiving PGE and oxygen supplementation (Table 2). Newborns treated with both PGE and oxygen had lower pre- and post-ductal saturation at 48 h (87.4 ± 7.3% (95% CI 85.0, 89.7) and 86.3 ± 7.0% (84.3, 88.3), respectively, *p* < 0.05) when compared to the other treatment categories (PGE only, oxygen only, or no support).

### 3.2. Secondary Analysis: Hypothetical Screening of Study Cohort

**Figure 3:**
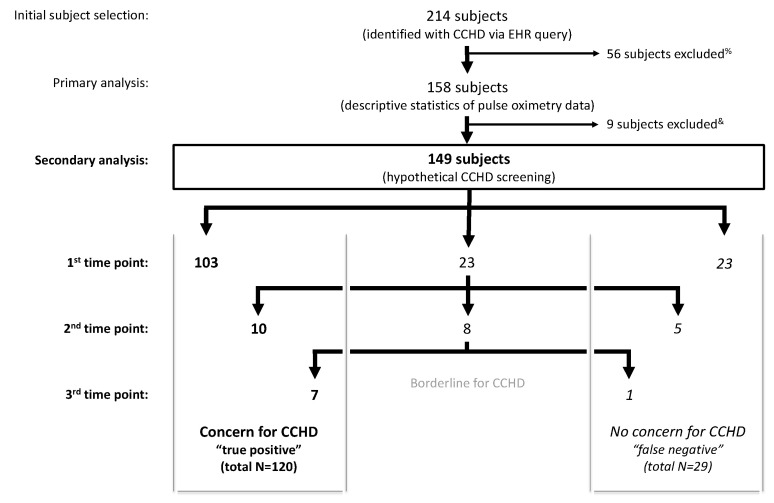
Application of the American Academy of Pediatrics (AAP) protocol to retrospective data in newborns with known CCHD (*n* = 149) results in 120 true positive screens and 80.5% sensitivity. ^%^ Exclusion criteria: insufficient pulse oximetry data in the electronic health record (EHR), death prior to 24 h of life, atrial septostomy or surgery prior to 24 h of life. ^&^ Inadequate number of pulse oximetry readings to complete the AAP protocol.

Application of the AAP screening cutoffs to the retrospective data independently at each time point (not allowing for rescreens) would have resulted in 67% failing a screen at 24 h (75 of 112 subjects), 66.7% at 28 h (76 of 114 subjects), 64.9% at 36 h (85 of 131 subjects), and 48.6% at 48 h (69 of 142 subjects). A sequential screening analysis, similar to the AAP protocol, was subsequently applied to 149 subjects as 9 of the 158 subjects were excluded due to an inadequate number of pulse oximetry readings to apply the AAP protocol (Figure 3). Twenty-nine subjects who screened negative with passing pulse oximetry readings resulted in a 19.5% hypothetical false negative screening rate in our cohort. The 29 false negative screened subjects represented all diagnosis categories. The false negative screening rate was 18.8% (*n* = 80) in subjects with a prenatal concern for CCHD and 20.3% (*n* = 69) in subjects without a prenatal concern for CCHD.

## 4. Discussion

In this study, we report mean pulse oximetry readings in a cohort of newborns with CCHD at altitude and thereby demonstrate the expected hypoxemia of the seven targeted CCHD diagnoses. Prior studies of newborn oxygen saturations have suggested a wider range and lower average saturations for infants born at higher altitude compared to those born at sea level [3,13,15]. We demonstrate in our cohort of infants at moderate altitude with the seven CCHD diagnoses targeted by the AAP (HLHS, TGA, TA, TAPVR, TVA, PA/IVS), that the mean pulse oximetry values are lower than recommended cutoffs for newborn screening. Wright and colleagues [3] evaluated the pulse oximetry screening protocol at 5557 feet (1694 m) and reported a mean pre-ductal saturation of 97.2% (±1.9) and a post-ductal saturation of 97.2% (±2.1) in 1003 healthy newborns at approximately 24 h of life (23.8 ± 2.3 h of life, mean ± SD), similar to what would be expected in a healthy newborn cohort at sea level and significantly higher than our cohort of infants with CCHD. Another study performed at a similar altitude demonstrated lower mean saturations of 92% and 93%, at 24 and 48 h, respectively [12]. Additionally, despite similar average saturation measurements, Wright et al. demonstrated a higher rate of screen failure. This variability certainly supports the need for further study of oxygen saturation and the screening protocol at moderate altitude.

In a secondary analysis we evaluated the hypothetical effectiveness of a sequential pulse oximetry screening protocol similar to the AAP-recommended protocol by application in a population of infants with known CCHD. By evaluating a cohort with known CCHD, we were able to demonstrate a 19.5% false negative screening rate, or a true positive screen capture rate of 80.5%, at moderate altitude. Ailes and colleagues sought to address the difficulties in studying this rare disease by simulation of the population [17] and we present an alternative strategy that yields comparable findings. Our findings are comparable to the true positive screening rate found by Thangaratinam and colleagues [18], who performed a meta-analysis in 2012 of 13 studies at sea level evaluating pulse oximetry screening and calculated an overall sensitivity of 76.5% in screening for CCHD. Further, our retrospective evaluation revealed a similar sensitivity to that of two other large prospective studies (65.5% and 75%) [19,20]. Notably, the retrospective study design allowed us to perform this evaluation in a relatively large cohort of patients with CCHD that has not yet been achievable with a prospective study design [18].

Evaluations of pulse oximetry screening of neonates supported in the intensive care unit have raised concerns for increased false positive rates, issues with oxygen supplementation, and the appropriate timing of screening [21,22,23,24]. Additionally, a study performed by Lueth and colleagues evaluated a modification to the AAP-recommended screening protocol with transient oxygen supplementation in newborns at moderate altitude. Their intent was to replicate sea-level atmospheric oxygen tension and induce pulmonary vasodilatation to potentiate the neonatal transition [11]. This group found an increase in specificity of screening with a reduction in false positive screens by providing transient supplemental oxygen to newborns with an indeterminate or borderline first screen at moderate altitude [11]. Because low oxygen saturation in CCHD is primarily caused by shunting of de-oxygenated blood to the systemic circulation, oxygen supplementation in clinical practice does not typically normalize the percent saturation to greater than 95% in hypoxemic CCHD. It is difficult to know to what degree oxygen supplementation resulted in an increase in pulse oximetry reading in our study; however, we found no difference in mean oxygen saturation between patients with CCHD treated with supplemental oxygen versus those without supplemental oxygen. Nevertheless, Table 2 shows the range of saturations in both subjects receiving oxygen and those with no support exceeding 95%. Of the 29 false negative subjects in our study, 15 received supplemental oxygen at one or more pulse oximetry readings (14 did not receive oxygen supplementation). It is certainly possible that removal of oxygen support in the subjects in our study with CCHD would induce further hypoxemia and increase the specificity of the screening test, however, we are unable to make this assessment from the data gathered. This question requires further investigation and the effect of oxygen supplementation on the screening protocol remains unclear. Our mean saturation data do provide some support to the findings of Lueth and colleagues suggesting that oxygen supplementation may not have a negative impact on the sensitivity of pulse oximetry screening for CCHD in newborns. We would not advocate for screening of hypoxemic newborns on oxygen supplementation in the intensive care unit at this time as this is not the intent of the screening guideline [6], however, the findings of this study may inform discussion regarding the timing and process for screening of well infants born at moderate-high altitude who frequently require supplemental oxygen therapy in the first days and weeks of life [13,25]. Certainly, any consideration of pulse oximetry screening for CCHD in the context of supplemental oxygen should be taken with scrutiny both of the patient physiology and use of the screening protocol guideline.

Our retrospective application of the AAP screening protocol did not allow us to exactly replicate the timing of the AAP screening protocol (three screens separated by 1 h each). In addition, the AAP screening protocol is meant to evaluate asymptomatic newborns not presenting in the first 24 h of life, whereas we studied newborns diagnosed with known CCHD. We identify both of these as limitations to this study, however, we used this cohort of infants with known CCHD to estimate the sensitivity of the screening protocol. Further, our retrospective approach resulted in 158 subjects while the very large prospective studies yielded 24–29 subjects with congenital heart disease after screening 20,000–40,000 newborns [18]. Certainly, a future direction will be to apply this study design to a larger cohort of infants with CCHD. An additional limitation is that we found a difference in CCHD diagnoses between the subjects included in the study when compared to those excluded. A disproportionate number of the subjects with transposition of the great arteries were excluded because of atrial septostomy performed before 24 h of life (commonly required for infants with TGA). This may suggest that our cohort of subjects is not representative of the general population of newborns with CCHD, however, newborns requiring atrial septostomy early in life are more likely to present in extremis and not require pulse oximetry screening to identify disease. Finally, this retrospective assessment was dependent on the EHR with pulse oximetry readings that were subject to measurement and recording error from the bedside care provider. These values were not taken for formal screening or research purposes, rather, the readings were collected as a part of routine clinical care. However, these subjects all received hourly oximetry readings recorded in the EHR and the standard is for validation and confirmation by a bedside nurse with interpretation of the waveform when the infants were calm. We identify this limitation to the study design and that of measurement and documentation error by the clinical care team in the EHR. Finally, we applied the screening protocol to newborns who received PGE, oxygen, or both PGE and oxygen, to allow comparison with those who did not receive either PGE or oxygen. We recognize the contribution of both PGE and supplemental oxygen in the interpretation of subjects’ pulse oximetry data; however, our results suggest that even when oxygen and PGE were administered the infants still had pulse oximetry values that were low and the therapy did not change the outcome of screening with pulse oximetry in these newborns.

We present the first report evaluating the oxygen saturations of a cohort of infants with known CCHD at moderate altitude. Additionally, we present the results of a hypothetical AAP-recommended pulse oximetry screening protocol in this same cohort. We also found that oxygen supplementation did not impact the ability of the screening protocol to identify CCHD in this cohort, which adds to the consideration of screening in neonatal intensive care units and at higher altitudes. We cannot assess the false positive rate of screening at moderate altitude with our cohort and encourage continued efforts for large population-based studies to evaluate pulse oximetry screening for CCHD.

## Figures and Tables

**Table 1:** Baseline subject characteristics (*n* = 214).

Characteristics	Study Subjects (*n* = 158)	Excluded Subjects (*n* = 56)	*p*-Value
Gestational age, weeks (range)	38.2 ± 2.4 (29–45)	38.3 ± 2.4 (30–41.3)	0.379
Birth weight, *g* (range)	2988.7 ± 579.3 (1230–4165)	3071.0 ± 671.7 (960–4780)	0.277
Male gender, *n* (%)	101 (63.9)	35 (62.5)	0.849
Race, *n* (%)			0.470
White/Caucasian	111 (70.3)	37 (66.1)	
Black/African American	10 (6.3)	3 (5.4)	
Hawaiian/Pacific Islander	1 (0.6)	0 (0)	
Asian	1 (0.6)	1 (1.8)	
American Indian/Alaskan Native	3 (3.2)	1 (1.8)	
Other	26 (16.5)	9 (16.1)	
Not specified	4 (2.5)	5 (8.9)	
Ethnicity, *n* (%)			0.088
Hispanic/Latino	54 (34.2)	15 (26.8)	
Not Hispanic/Latino	100 (63.3)	36 (64.3)	
Not specified	4 (2.5)	5 (8.9)	
Apgar score, score (range)			
1 min	7 ± 2 (0–9)	7 ± 2 (2–8)	0.409
5 min	8 ± 1 (0–9)	8 ± 1 (4–9)	0.212
Prenatal diagnosis of CCHD, *n* (%)	84 (53.2)	20 (35.7)	0.152
Family history of CCHD, *n* (%)	18 (11.4)	3 (5.4)	0.361
Genetic diagnosis, *n* (%)			0.825
Down syndrome	2 (1.3)	0 (0)	
22q11 deletion	6 (3.8)	1 (1.8)	
None specified	150 (94.9)	50 (89.3)	
CCHD diagnosis, *n* (%)			0.002 *
HLHS	48 (30.4)	17 (30.4)	
TOF	34 (21.5)	5 (8.9)	
TGA	30 (19.0)	26 (46.4)	
TA	13 (8.2)	2 (3.6)	
TAPVR	10 (6.3)	5 (8.9)	
TVA	10 (6.3)	0 (0)	
PA/IVS	7 (4.4)	1 (1.8)	
Combination of above	6 (3.8)	0 (0)	
Maternal age, years (range)	28.1 ± 5.8 (15–42)	26.5 ± 6.0 (15–41)	0.089
Maternal diabetes status, *n* (%)			0.272
Diabetes (Type I or Type II)	7 (4.5)	0 (0)	
Gestational diabetes	6 (3.8)	3 (5.4)	
Not diabetic	144 (91.7)	53 (94.6)	

Diagnoses are abbreviated: HLHS, hypoplastic left heart syndrome; TOF, tetralogy of Fallot; TGA, transposition of the great arteries; TA, truncus arteriosus; TAPVR, total anomalous pulmonary venous return; TVA, tricuspid valve atresia; PA/IVS, pulmonary valve atresia with intact ventricular septum; CCHD, critical congenital heart disease. * *p* < 0.05.

**Table 2:** Pre- and post-ductal pulse oximetry readings, PGE administration, and oxygen supplementation data at 24, 28, 36, and 48 h of life (*n* = 158). * *p* < 0.05.

		No Support	PGE	Oxygen	Both PGE and Oxygen
**24-hours**	**Pre-ductal**	90.3 ± 7.7 (77–100) (*n* = 7)	88.6 ± 7.3 (75–100) (*n* = 30)	86.6 ± 4.3 (80–95) (*n* = 14)	85.2 ± 7.6 (70–98) (*n* = 36)
	**Post-ductal**	89.4 ± 5.4 (80–97) (*n* = 14)	87.8 ± 6.3 (73–99) (*n* = 33)	88.9 ± 6.0 (78–97) (*n* = 18)	86.8 ± 6.6 (67–100) (*n* = 42)
**28-hours**	**Pre-ductal**	88.0 ± 5.0 (81–95) (*n* = 7)	88.3 ± 6.8 (74–100) (*n* = 28)	84.6 ± 7.1 (72–96) (*n* = 18)	86.1 ± 7.8 (53–96) (*n* = 34)
	**Post-ductal**	90.3 ± 6.2 (78–100) (*n* = 14)	88.5 ± 7.4 (70–100) (*n* = 35)	85.7 ± 6.7 (72–95) (*n* = 20)	86.2 ± 7.6 (68–100) (*n* = 43)
**36-hours**	**Pre-ductal**	89.0 ± 9.2 (77–99) (*n* = 4)	88.4 ± 5.0 (76–100) (*n* = 33)	86.3 ± 8.0 (65–98) (*n* = 16)	85.5 ± 8.9 (65–100) (*n* = 38)
	**Post-ductal**	90.0 ± 4.9 (82–100) (*n* = 15)	90.2 ± 4.4 (79–98) (*n* = 46)	88.9 ± 7.0 (72–100) (*n* = 22)	86.1 ± 8.2 (68–99) (*n* = 46)
**48-hours**	**Pre-ductal**	91.0 ± 5.3 (80–97) (*n* = 8)	90.4 ± 6.4 (70–100) (*n* = 42)	89.8 ± 4.5 (81–98) (*n* = 13)	87.4 ± 7.3 * (67–100) (*n* = 35)
	**Post-ductal**	90.3 ± 6.4 (75–100) (*n* = 21)	90.5 ± 5.2 (75–99) (*n* = 51)	91.5 ± 4.8 (80–97) (*n* = 20)	86.3 ± 7.0 * (63–100) (*n* = 45)
